# Master regulators of wood formation in *Eucalyptus*

**DOI:** 10.1186/1753-6561-5-S7-P110

**Published:** 2011-09-13

**Authors:** Hua Wang, Marcal Soler, Hong Yu, Eduardo Leal Oliveira Camargo, Hélène San Clemente, Bruno Savelli, Nathalie Ladouce, Jorge Paiva, Jacqueline Grima-Pettenati

**Affiliations:** 1 LRSV, UMR 5546 Université Toulouse III /CNRS, BP 42617, 31326 Castanet-Tolosan, France; 2 LRSV, UMR 5546 Université Toulouse III /CNRS, BP 42617, 31326 Castanet-Tolosan, France//LGE, IB, UNICAMP, Campinas, Sao Paulo, Brazil; 3 IICT/MCTES - Palácio Burnay - Rua da Junqueira, 30, 1349-007 Lisboa, Portugal

## 

With the current global focus on bioenergy, forest plantations are increasingly becoming important sources for second generation biofuel, where the whole plant lignocellulosic biomass is to be mobilized. The lignocellulosic biomass is mainly composed of secondary walls (SW) possessing unique characteristics (biochemical composition and tridimensional association of polymers), which govern the intrinsic properties of wood. They especially contain high amounts of lignins, hydrophobic phenolic polymers which constitute an obstacle to the optimal utilization of plant species in paper industry and for saccharification prior to bioethanol production. Among perennial species, *Eucalyptus* species grow very fast and produce high yields of lignocellulosic biomass. They represent the main industrial plantations in the worldand one of the most appealing lignocellulosic feedstock for bioenergy production. Dissection of the molecular switches controlling the coordinated lignin biosynthetic genes is therefore of utmost importance to understand the molecular mechanisms underlying tissue specific deposition of lignin and be able to improve secondary cell wall properties.

With the objective of improving Eucalyptus wood quality to better-fit industrial applications, we are focusing our efforts towards the identification and functional characterisation of regulatory genes controlling the biosynthesis of the cell wall polymers (mainly lignins).

We performed a precise mapping and functional characterization of the *cis*-regulatory elements contained in the promoters of two genes encoding key and consecutive steps of the lignin biosynthetic pathway *i.e.* Cinnamoyl CoA reductase (CCR) and Cinnamyl Alcohol dehydrogenase (CAD) (Rahantamala *et al*, 2010). Our results supported a major role for the MYB transcription factors (TF) consensus sites in the control of the coordinated expression of these two genes. The functional analysis of two MYB factors (EgMYB1 and EgMYB2) preferentially expressed in *Eucalyptus* xylem revealed that they are able to bind specifically to these promoters and regulate transcription*in vivo*. EgMYB1 behaves as a repressor whereas EgMYB2 is an activator (Goicoechea *et al*, 2005, Legay et al, 2010) of the lignin biosynthetic genes but also of the secondary wall biosynthesis. Indeed, both MYBs were shown recently to be master genes regulating the entire secondary wall biosynthetic program including cellulose, xylan and lignin genes (Zhong *et al*, 2010; Legay *et al* 2010). The presence of both positive and negative regulators in *Eucalyptus* xylem offers the possibility of a combinatorial control of gene expression that could provide the necessary flexibility to ensure tight temporal and spatial regulation of lignin biosynthesis or secondary cell wall.

To address this question and get a deeper insight the complex regulation of the SW formation in *Eucalyptus*, we are now studying the regulation of these two MYBs including fine spatial and temporal expression, identification of their direct targets genes and of their protein partners. We have constructed a yeast-two-hybrid library from *Eucalyptus* xylem that will also be instrumental for deciphering the interactants of landmark genes for the International *Eucalyptus* community. Thanks to the recent release of the *E. grandis* genome (*Eucalyptus grandis* Genome Project 2010, http://www.phytozome.net/eucalyptus), we have performed a genome-wide survey of the large R2R3-MYB superfamily. The phylogenetic comparison of this family with *Arabidopsis*, rice, poplar and grapevine showed a marked expansion of some clusters putatively involved in wood-related processes. Some R2R3 MYB genes seem to be specific of woody plants. The spatiotemporal expression patterns of members of such clusters are currently being studied. Although Auxin is known as a key regulator of cambium activity and wood formation, the Auxin response mediators [Auxin/Indole-3-Acetic Acid (Aux/IAA) and Auxin Response Factor (ARF) transcription factors] extensively characterized in model plants, are still largely uncharacterized in tree species. We have identified 23 *Aux/IAA* and 17 *ARF* in the *E. grandis* genome. Comparative phylogenetic analysis revealed that several *Aux/IAA* and *ARF* subgroups have differentially expanded or contracted amongst the three dicotyledonous plants studied (*Arabidopsis*, *Populus* and *Eucalyptus*). Expression analysis and EST database surveys are currently underway to explore the transcript levels of each member in the different organs and tissues of *Eucalyptus* at key developmental stages as well as in response to hormonal treatments and to environmental stresses. Further functional genomics studies conducted on new candidate transcription factors, their regulation under the developmental or environmental stimuli will help identifying major factors underpinning the physicochemical properties of cell walls, the recalcitrance of which remains a key scientific challenge for establishing highly efficient, sustainably produced, second-generation biofuels.

